# Genetic effects of *FASN*, *PPARGC1A*, *ABCG2* and *IGF1* revealing the association with milk fatty acids in a Chinese Holstein cattle population based on a post genome-wide association study

**DOI:** 10.1186/s12863-016-0418-x

**Published:** 2016-07-28

**Authors:** Cong Li, Dongxiao Sun, Shengli Zhang, Shaohua Yang, M. A. Alim, Qin Zhang, Yanhua Li, Lin Liu

**Affiliations:** 1 Department of Animal Genetics and Breeding, College of Animal Science and Technology, Key Laboratory of Animal Genetics and Breeding of Ministry of Agriculture, National Engineering Laboratory for Animal Breeding, China Agricultural University, 2 Yuanmingyuan West Road, Beijing, 100193 China; 2 Beijing Dairy Cattle Center, Beijing, 100085 China

**Keywords:** Association analysis, Candidate gene, Haplotype, Milk fatty acids, Single nucleotide polymorphism

## Abstract

**Background:**

A previous genome-wide association study deduced that one (ARS-BFGL-NGS-39328), two (Hapmap26001-BTC-038813 and Hapmap31284-BTC-039204), two (Hapmap26001-BTC-038813 and BTB-00246150), and one (Hapmap50366-BTA-46960) genome-wide significant single nucleotide polymorphisms (SNPs) associated with milk fatty acids were close to or within the *fatty acid synthase* (*FASN*), *peroxisome proliferator-activated receptor gamma, coactivator 1 alpha* (*PPARGC1A*), *ATP-binding cassette, sub-family G, member 2* (*ABCG2*) and *insulin-like growth factor 1* (*IGF1*) genes. To further confirm the linkage and reveal the genetic effects of these four candidate genes on milk fatty acid composition, genetic polymorphisms were identified and genotype-phenotype associations were performed in a Chinese Holstein cattle population.

**Results:**

Nine SNPs were identified in *FASN*, among which SNP rs41919985 was predicted to result in an amino acid substitution from threonine (ACC) to alanine (GCC), five SNPs (rs136947640, rs134340637, rs41919992, rs41919984 and rs41919986) were synonymous mutations, and the remaining three (rs41919999, rs132865003 and rs133498277) were found in *FASN* introns. Only one SNP each was identified for *PPARGC1A*, *ABCG2* and *IGF1*.

Association studies revealed that *FASN*, *PPARGC1A*, *ABCG2* and *IGF1* were mainly associated with medium-chain saturated fatty acids and long-chain unsaturated fatty acids, especially *FASN* for C10:0, C12:0 and C14:0. Strong linkage disequilibrium was observed among ARS-BFGL-NGS-39328 and rs132865003 and rs134340637 in *FASN* (D´ > 0.9), and among Hapmap26001-BTC-038813 and Hapmap31284-BTC-039204 and rs109579682 in *PPARGC1A* (D´ > 0.9). Subsequently, haplotype-based analysis revealed significant associations of the haplotypes encompassing eight *FASN* SNPs (rs41919999, rs132865003, rs134340637, rs41919992, rs133498277, rs41919984, rs41919985 and rs41919986) with C10:0, C12:0, C14:0, C18:1n9c, saturated fatty acids (SFA) and unsaturated fatty acids (UFA) (*P* = 0.0204 to *P* < 0.0001).

**Conclusion:**

Our study confirmed the linkage between the significant SNPs in our previous genome-wide association study and variants in *FASN* and *PPARGC1A*. SNPs within *FASN*, *PPARGC1A*, *ABCG2* and *IGF1* showed significant genetic effects on milk fatty acid composition in dairy cattle, indicating their potential functions in milk fatty acids synthesis and metabolism. The findings presented here provide evidence for the selection of dairy cows with healthier milk fatty acid composition by marker-assisted breeding or genomic selection schemes, as well as furthering our understanding of technological processing aspects of cows’ milk.

**Electronic supplementary material:**

The online version of this article (doi:10.1186/s12863-016-0418-x) contains supplementary material, which is available to authorized users.

## Background

Recently, an increasing number of genes have been reported as associated with milk production for dairy cattle breeding, and great improvements have been obtained. Many quantitative trait locus (QTL) analysis and association studies revealed the *DGAT1*, *GHR*, *FASN* and *PPARGC1A* genes as promising candidate genes for milk production traits [1–12]. Nevertheless, there have been few reports [13–22] of association studies involving milk fatty acid traits, which should be considered because of their close relation with milk flavor and nutritional properties. High concentrations of saturated fatty acids (SFAs) such as C12:0, C14:0 and C16:0 increase the risks of coronary artery disease (CAD) by promoting the concentrations of blood low density lipoprotein (LDL) cholesterol [23], while polyunsaturated fatty acids (PUFAs) have the ability to reduce blood fat and cholesterol levels by inhibiting fat formation and enzyme activities acting on fat [24, 25]. Thus, increasing the ratio of PUFAs to SFAs would be beneficial to human health. A previous genome-wide association study (GWAS) revealed that several significant single nucleotide polymorphisms (SNPs) close to or within the *FASN*, *PPARGC1A*, *ABCG2* and *IGF1* genes were associated with milk fatty acids in Chinese Holstein dairy cattle [26]. In addition, the *FASN*, *PPARGC1A*, *ABCG2* and *IGF1* genes were observed to be associated significantly with milk production traits in our previous candidate genes analysis in Chinese Holstein cattle [27–30]. Therefore, we deduced that the significant SNPs might be linked with the causative mutations in these four genes. The purpose of the present study was to identify the genetic effects of the *FASN*, *PPARGC1A*, *ABCG2* and *IGF1* genes on traits of milk fatty acids in a Chinese Holstein cattle population. In addition, linkage disequilibrium (LD) analyses were conducted among the SNPs identified in our previous GWAS and in this study.

## Methods

### Phenotypic data and traits

Complete details of the milk sample collection and the detection method for milk fatty acids have been reported previously [26]. Briefly, fat was extracted from 2 mL of milk and then methyl esterification of fats was performed. One milliliter of methyl esters of fatty acids were prepared and determined by gas chromatography using a gas chromatograph (6890 N, Agilent) equipped with a flame-ionization detector and a high polar fused silica capillary column (SPTM-2560, 100 m × 0.25 mm ID, 0.20 μm film; Cat. No. 24056). About 1 μL of the sample was injected under the specific gas chromatography conditions. Finally, individual fatty acids were identified and quantified by comparing the methyl ester chromatograms of the milk fat samples with the chromatograms of pure fatty acids methyl ester standards (SupelcoTM 37 Component FAME Mix), and were measured as the weight proportion of total fat weight (wt/wt%). Phenotypic values of 10 main milk fatty acids were tested directly using gas chromatography, which included SFAs of C10:0, C12:0, C14:0, C16:0, C18:0, monounsaturated fatty acids (MUFAs) of C14:1, C16:1, C18:1n9c, and PUFAs of CLA (cis-9, trans-11 C18:2), C18:2n6c. Based on the phenotypes of 10 tested milk fatty acids, six additional traits were obtained including SFA, UFA, SFA/UFA (the ratio of SFA to UFA), C14 index, C16 index and C18 index. The three indices were calculated as $\frac{\mathrm{cis}\hbox{-} 9\kern0.5em \mathrm{unsaturated}}{\mathrm{cis}\hbox{-} 9\kern0.5em \mathrm{unsaturated}+\mathrm{saturated}}\ast 100$, [31].

The population in this study comprised 346 Chinese Holstein cows, which were the daughters of 13 sire families from 13 farms of the Beijing Sanyuan Dairy Farm Center. Sixteen main milk fatty acid traits were considered in this association study.

### Genomic DNA extraction

The whole blood samples corresponding to the 346 Chinese Holstein cows with phenotypic values were collected. Genomic DNA was extracted from blood samples of the cows using a TIANamp Genomic DNA kit (TianGen, Beijing, China) according to the manufacturer’s instructions and frozen semen of the sires using a standard phenol-chloroform procedure. The quantity and quality of the extracted DNA were measured using a NanoDrop™ ND-2000c Spectrophotometer (Thermo Scientific, Inc.) and by gel electrophoresis.

### SNP identification and genotyping

A DNA pool was constructed from aforementioned 13 Holstein bulls (50 ng/μL for each individual) whose daughters were used for the association analysis to identify potential SNPs in the *FASN*, *PPARGC1A*, *ABCG2* and *IGF1* genes. For *FASN*, a total of 30 pairs of PCR primers (Additional file 1, Table S1) were designed to amplify all the exons and their partial flanking intronic sequences based on the reference sequence of the bovine *FASN* referring to Bos_taurus_UMD_3.1 assembly (NCBI Reference Sequence: AC_000176.1) using Primer3 web program (v.0.4.0) [32]. Following with the same method, a pair of specific primers was designed for selective amplification based on the exon 9 and partial intron 9 sequence of *PPARGC1A* (NCBI Reference Sequence: AC_000163.1): forward 5′- GCC GGT TTA TGT TAA GAC AG-3′ and reverse 5′- GGT ATT CTT CCC TCT TGA GC-3′. Primers were also designed from exon 7 and partial flanking intronic sequences of the *ABCG2* gene (NCBI Reference Sequence: AC_000163.1): forward 5′- TAA AGG CAG GAG TAA TAA AG-3′ and reverse 5′- TAA CAC CAA ACT AAC CGA AG-3′, and the 5′-flanking region of the *IGF1* gene (NCBI Reference Sequence: AC_000162.1): forward 5′- ATT ACA AAG CTG CCT GCC CC-3′ and reverse 5′- CAC ATC TGC TAA TAC ACC TTA CCC G-3′.

Polymerase chain reaction (PCR) amplifications for the pooled DNA from the 13 sires were performed in a final reaction volume of 25 μL comprising of 50 ng of genomic DNA, 0.5 μL of each primer (10 mM), 2.5 μL of 10 × PCR buffer, 2.5 mM each of dNTPs, and 1 U of Taq DNA polymerase (Takara, Dalian, China). The PCR protocol was 5 min at 94 °C for initial denaturation followed by 34 cycles at 94 °C for 30 s; 56 ~ 60 °C for 30 s; 72 °C for 30 s; and a final extension at 72 °C for 7 min for all primers. The PCR products were purified to remove residual primers, dNTPs and reagents from the amplification reaction. A gel purification kit (DNA Gel Extraction Kit, TransGen Biotech, China) was used to extract the target DNA band. Then, 15 μL of each purified PCR product with 1 μL of each forward and reverse primer, was bi-directionally sequenced using an ABI3730XL sequencer (Applied Biosystems, Foster City, CA, USA).

Matrix-assisted laser desorption/ionization time of flight mass spectrometry (MALDI-TOF MS, Sequenom MassARRAY, Bioyong Technologies Inc. HK) was used for subsequent genotyping of the 346 Chinese Holstein cows.

### Linkage disequilibrium (LD) analysis and haplotype construction

Pair-wise LD was measured between the genotyped SNPs of each gene and the corresponding adjacent SNPs that were significantly associated with target traits identified in our previous GWAS based on the criterion of D’ using the software Haploview [33]. Accordingly, haplotype blocks where SNPs are in high LD (D’ > 0.90) were also determined based on confidence interval methods [34]. A haplotype with a frequency >5 % was treated as a distinguishable haplotype, and those haplotypes each with relative frequency <5 % were pooled into a single group.

### Association analyses

Hardy-Weinberg equilibrium tests were performed on each identified SNP. A goodness-of-fit test (Chi-square) was used to compare the number of expected and observed genotypes, using 0.05 as significant threshold value.

The mixed procedure of SAS 9.3 software (SAS Institute Inc., Cary, NC) with the following animal model was performed to estimate the genetic effects of each candidate SNP or haplotype on the milk fatty acid traits.

$$ {y}_{i\mathrm{jklmn}}=\mu +{\mathrm{F}}_{\mathrm{i}}+{\mathrm{P}}_{\mathrm{j}}+{L}_k+{G}_l+{\alpha}_m+{e}_{ijklmn} $$

where, y_ijklmn_ was the phenotypic value of each trait of the cows; μ was the overall mean; F_i_ was the fixed effect of the farm; P_j_ was the fixed effect of parity; L_k_ was the fixed effect of the stage of lactation; G_l_ was the fixed effect corresponding to the genotype of polymorphisms or haplotype; α_m_ was the random polygenic effect, distributed as N (0, Aσ_a_^2^), with the additive genetic relationship matrix A and the additive genetic variance σ_a_^2^; and e_ijklmn_ was the random residual, distributed as N (0, Iσ_e_^2^), with identity matrix I and residual error variance σ_e_^2^. Bonferroni correction was adopted to correct for multiple testing. The significance level of the multiple tests was equal to the raw *P* value divided by number of tests. In the present study, three genotypes were compared for each trait mean that three multiple comparisons needed to be performed, therefore, Bonferroni corrected significance levels of 0.05/3 = 0.0167 and 0.01/3 = 0.0033 were used. For the haplotype, the Bonferroni corrected significance levels were presented as 0.05/N, where N refers to the number of formed haplotypes. The additive (a), dominance (d) and allele substitution (α) effects were estimated according to the equation proposed by Falconer & Mackay [35], i.e. $$ \mathrm{a}=\raisebox{1ex}{$\left(\mathrm{AA}-\mathrm{B}\mathrm{B}\right)$}\!\left/ \!\raisebox{-1ex}{$2$}\right. $$, $$ \mathrm{d}=\mathrm{AB}-\raisebox{1ex}{$\left(\mathrm{AA}+\mathrm{B}\mathrm{B}\right)$}\!\left/ \!\raisebox{-1ex}{$2$}\right. $$ and α = a + d(q − p), where AA and BB represent the two homozygous genotypes, AB is the heterozygous genotype, and p and q are the allele frequencies of the corresponding alleles.

## Results

### SNPs identification

After sequencing the PCR products directly using the pooled genomic DNA, a total of nine SNPs were identified for the *FASN* gene. Of these, three were located in the intronic region and six were in exons. The SNP in exon 39 (rs41919985) was predicted to result in an amino acid replacement (A2266T) from threonine (ACC) to alanine (GCC) in the FASN protein, and the other five SNPs in the coding region (rs136947640, rs134340637, rs41919992, rs41919984 and rs41919986) were synonymous mutations. Regarding *PPARGC1A*, *ABCG2* and *IGF1*, only one SNP was detected in each gene (rs109579682, rs137757790 and rs109763947, respectively), of which rs109763947 is located in the 5′-untranslated region (UTR) and the other two SNPs are in intronic regions. The detailed SNP information is shown in Table 1, and the five significant SNPs for milk fatty acids that are close to *FASN*, *PPARGC1A*, *ABCG2* and *IGF1* identified in our previous GWAS [26] are listed as well. All the identified SNPs in this study were found to be in Hardy-Weinberg equilibrium (*P* > 0.01, Tables 2 and 3).

**Table 1:** SNPs information identified in this study and in a previous GWA study

CHR	RefSNP	Locus	Allele	Gene region	Position^a^	Amino acid substitution	Gene	Origin
5	rs109763947	g.1407C > T	C/T	5'-UTR	66605011		*IGF1*	This study
5	rs41643203	Hapmap50366-BTA-46960	C/T	intron-2	68610818		Close to *IGF1*	[23]
6	rs109579682	g.85330C > T	C/T	Intron-9	44875251		*PPARGC1A*	This study
6	rs110131167	Hapmap26001-BTC-038813	A/G	intron-2	44926243		*PPARGC1A*	[23]
6	rs108967640	Hapmap31284-BTC-039204	C/T	-	45096462		*PPARGC1A*	[23]
6	rs137757790	g.45599A > C	A/C	Intron-7	38005668		*ABCG2*	This study
6	rs43450879	BTB-00246150	A/G	Intron-1	20993424		Close to *ABCG2*	[23]
19	rs136947640	g.7709 T > C	T/C	Exon-10	51391830		*FASN*	This study
19	rs41919999	g.8948C > T	C/T	Intron-12	51393068		*FASN*	This study
19	rs132865003	g.10568 T > C	T/C	Intron-18	51394689		*FASN*	This study
19	rs134340637	g.11280G > A	G/A	Exon-21	51395400		*FASN*	This study
19	rs41919992	g.13965C > T	C/T	Exon-27	51398083		*FASN*	This study
19	rs133498277	g.14439 T > C	T/C	Intron-28	51398557		*FASN*	This study
19	rs41919984	g.16907 T > C	T/C	Exon-37	51401022		*FASN*	This study
19	rs41919985	g.17924A > G	A/G	Exon-39	51402032	A2266T	*FASN*	This study
19	rs41919986	g.18663 T > C	T/C	Exon-42	51402774		*FASN*	This study
19	rs41921177	ARS-BFGL-NGS-39328	A/G	Intron-11	51326750		Close to *FASN*	[23]

Note: ^a^All SNP nucleotide positions were derived from the Bos_taurus_UMD_3.1 assembly (GenBank accession number: AC_000171.1)

**Table 2:** Genotypic and allelic frequencies and Hardy-Weinberg equilibrium test of nine SNPs of the *FASN* gene in Chinese Holstein cattle

Position	Locus	Genotypes	*N*	Frequency	Allele	Frequency	Hardy-Weinberg equilibrium *χ*2 test
Exon-10	rs136947640	CC	248	0.790	C	0.892	*P* > 0.05
		TT	2	0.006	T	0.108	
		CT	64	0.204			
Intron-12	rs41919999	CC	64	0.204	C	0.462	*P* > 0.05
		TT	88	0.280	T	0.538	
		CT	162	0.516			
Intron-18	rs132865003	CC	220	0.698	C	0.833	*P* > 0.05
		TT	10	0.032	T	0.167	
		CT	85	0.270			
Exon-21	rs134340637	AA	10	0.032	A	0.167	*P* > 0.05
		GG	220	0.698	G	0.833	
		AG	85	0.270			
Exon-27	rs41919992	CC	157	0.500	C	0.712	*P* > 0.05
		TT	24	0.076	T	0.288	
		CT	133	0.424			
Intron-28	rs133498277	CC	157	0.500	C	0.713	*P* > 0.05
		TT	23	0.073	T	0.287	
		CT	134	0.427			
Exon-37	rs41919984	CC	157	0.498	C	0.711	*P* > 0.05
		TT	24	0.076	T	0.289	
		CT	134	0.425			
Exon-39	rs41919985	AA	25	0.079	A	0.290	*P* > 0.05
		GG	157	0.498	G	0.710	
		AG	133	0.422			
Exon-42	rs41919986	CC	155	0.497	C	0.708	*P* > 0.05
		TT	25	0.080	T	0.292	
		CT	132	0.423

**Table 3:** Genotypic and allelic frequencies and Hardy-Weinberg equilibrium test of SNPs of the *PPARGC1A, ABCG2 and IGF1* genes in Chinese Holstein cattle

Gene	Position	Locus	Genotypes	*N*	Frequency	Allele	Frequency	Hardy-Weinberg equilibrium *χ*2 test
*PPARGC1A*	Intron-9	rs109579682	CC	27	0.078	C	0.292	*P* > 0.05
			TT	170	0.494	T	0.708	
			CT	147	0.427			
*ABCG2*	Intron-7	rs137757790	AA	115	0.333	A	0.543	*P* > 0.01
			CC	85	0.246	C	0.457	
			AC	145	0.420			
*IGF1*	5’-UTR	rs109763947	CC	58	0.168	C	0.439	*P* > 0.05
			TT	100	0.290	T	0.561	
			CT	187	0.542			

### Associations between the four candidate genes and milk fatty acid traits

Associations between the nine SNPs of *FASN* and 16 milk fatty acid composition traits are presented in Table 4. We found that all nine SNPs showed significant associations with at least one milk fatty acid trait. Of these, three SNPs (rs136947640, rs132865003 and rs134340637) were only significantly associated with C18:2n6c (*P* < 0.0001, *P* = 0.0128, *P* = 0.0128), two SNPs (rs41919992 and rs133498277) showed strong associations with seven traits of C10:0, C12:0, C14:0, C18:1n9c, C16 index, SFA and UFA (*P* = 0.0190 to < 0.0001), three SNPs (rs41919984, rs41919985 and rs41919986) were strongly associated with the above seven traits plus SFA/UFA (*P* = 0.045 to *P* <0.0001), and one SNP (rs41919999) showed significant association with C10:0 (*P* = 0.0012), C12:0 (*P* = 0.0041) and C14:0 (*P* = 0.0071). Meanwhile, for C14:1, C16:0, C16:1, C18:0, CLA, C14 index and C18 index, no significant SNPs in *FASN* were detected. Furthermore, the results showed that heterozygous genotypes of these SNPs were the dominant type for saturated fatty acids (C10:0, C12:0, C14:0, SFA and SFA/UFA), and the homozygotic genotypes of these SNPs were dominant for unsaturated fatty acids (C18:1n9c, C16 index and UFA).

**Table 4:** Associations of nine SNPs of the *FASN* gene with milk medium-chain fatty acids (MCFAs) in Chinese Holstein cattle (LSM ± SE)

Locus	Genotypes	C10:0	C12:0	C14:0	C14:1		
rs136947640	CC(248)	2.13 ± 0.06	2.63 ± 0.08	9.55 ± 0.13	0.79 ± 0.03		
	TT(2)	2.23 ± 0.24	2.66 ± 0.32	8.92 ± 0.54	0.65 ± 0.16		
	CT(64)	2.09 ± 0.07	2.56 ± 0.09	9.42 ± 0.15	0.78 ± 0.04		
	*P-value*	0.6139	0.5290	0.2611	0.6836		
rs41919999	CC(64)	2.13 ± 0.07^AB^	2.68 ± 0.09^AB^	9.64 ± 0.15^A^	0.76 ± 0.04		
	TT(88)	1.99 ± 0.07^B^	2.53 ± 0.09^B^	9.25 ± 0.14^B^	0.79 ± 0.04		
	CT(162)	2.15 ± 0.06^A^	2.73 ± 0.08^A^	9.52 ± 0.13^A^	0.80 ± 0.03		
	*P-value*	0.0012	0.0041	0.0071	0.6264		
rs132865003	CC(220)	2.11 ± 0.06	2.68 ± 0.08	9.52 ± 0.13	0.80 ± 0.03		
	TT(10)	2.17 ± 0.12	2.72 ± 0.16	9.45 ± 0.26	0.75 ± 0.08		
	CT(85)	2.11 ± 0.06	2.68 ± 0.08	9.49 ± 0.14	0.78 ± 0.04		
	*P-value*	0.8601	0.9610	0.9217	0.7157		
rs134340637	AA(10)	2.17 ± 0.12	2.72 ± 0.16	9.45 ± 0.26	0.75 ± 0.08		
	GG(220)	2.11 ± 0.06	2.68 ± 0.08	9.52 ± 0.13	0.80 ± 0.03		
	AG(85)	2.11 ± 0.06	2.68 ± 0.08	9.49 ± 0.14	0.78 ± 0.04		
	*P-value*	0.8601	0.9610	0.9217	0.7157		
rs41919992	CC(157)	2.05 ± 0.06^A^	2.53 ± 0.08^A^	9.31 ± 0.13^A^	0.77 ± 0.04		
	TT(24)	2.06 ± 0.09^AB^	2.45 ± 0.12^A^	9.35 ± 0.20^A^	0.76 ± 0.06		
	CT(133)	2.20 ± 0.06^B^	2.74 ± 0.08^B^	9.75 ± 0.13^B^	0.79 ± 0.04		
	*P-value*	0.0013	<.0001	<.0001	0.6169		
rs133498277	CC(157)	2.05 ± 0.06^A^	2.53 ± 0.08^A^	9.32 ± 0.13^A^	0.77 ± 0.04		
	TT(23)	2.07 ± 0.09^AB^	2.47 ± 0.12^A^	9.42 ± 0.20^AB^	0.75 ± 0.06		
	CT(134)	2.18 ± 0.06^B^	2.73 ± 0.08^B^	9.75 ± 0.13^B^	0.79 ± 0.04		
	*P-value*	0.0043	0.0003	<.0001	0.6826		
rs41919984	CC(157)	2.06 ± 0.09^AB^	2.51 ± 0.11^A^	9.38 ± 0.19^AB^	0.76 ± 0.06		
	TT(24)	2.04 ± 0.06^B^	2.58 ± 0.08^A^	9.29 ± 0.13^B^	0.78 ± 0.04		
	TC(134)	2.19 ± 0.06^A^	2.81 ± 0.08^B^	9.74 ± 0.13^A^	0.80 ± 0.04		
	*P-value*	0.0010	<.0001	<.0001	0.6958		
rs41919985	AA(25)	2.08 ± 0.09^AB^	2.54 ± 0.11^A^	9.46 ± 0.19^AB^	0.76 ± 0.06		
	GG(157)	2.04 ± 0.06^B^	2.58 ± 0.08^A^	9.28 ± 0.13^B^	0.78 ± 0.04		
	GA(133)	2.18 ± 0.06^A^	2.80 ± 0.08^B^	9.73 ± 0.13^A^	0.80 ± 0.04		
	*P-value*	0.0017	<.0001	<.0001	0.7268		
rs41919986	CC(155)	2.03 ± 0.06^A^	2.54 ± 0.08^A^	9.36 ± 0.13^A^	0.77 ± 0.04		
	TT(25)	2.08 ± 0.09^AB^	2.50 ± 0.11^A^	9.50 ± 0.19^AB^	0.76 ± 0.06		
	CT(132)	2.17 ± 0.06^B^	2.74 ± 0.08^B^	9.78 ± 0.13^B^	0.79 ± 0.04		
	*P-value*	0.0015	0.0002	<.0001	0.7225		
Locus	Genotypes	C16:0	C16:1	C18:0	C18:1n9c	C18:2n6c	CLA
rs136947640	CC(248)	32.30 ± 0.33	1.75 ± 0.05	12.59 ± 0.17	29.36 ± 0.22	4.03 ± 0.03^A^	0.38 ± 0.01
	TT(2)	32.38 ± 1.52	1.86 ± 0.21	12.25 ± 0.85	30.18 ± 1.13	3.73 ± 0.13^A^	0.38 ± 0.05
	CT(64)	32.17 ± 0.40	1.81 ± 0.06	12.54 ± 0.21	29.36 ± 0.28	4.12 ± 0.03^B^	0.40 ± 0.01
	*P-value*	0.9155	0.3007	0.9028	0.7714	<.0001	0.3536
rs41919999	CC(64)	32.22 ± 0.40	1.79 ± 0.06	12.31 ± 0.21	29.43 ± 0.28	4.09 ± 0.03	0.40 ± 0.01
	TT(88)	32.23 ± 0.38	1.77 ± 0.05	12.68 ± 0.20	29.82 ± 0.27	4.08 ± 0.03	0.38 ± 0.01
	CT(162)	32.11 ± 0.34	1.76 ± 0.05	12.50 ± 0.17	29.36 ± 0.23	4.07 ± 0.03	0.39 ± 0.01
	*P-value*	0.8921	0.7478	0.2182	0.0982	0.5542	0.4618
rs132865003	CC(220)	32.24 ± 0.33	1.75 ± 0.05	12.54 ± 0.17	29.44 ± 0.22	4.06 ± 0.03^a^	0.38 ± 0.01
	TT(10)	32.65 ± 0.73	1.78 ± 0.10	12.43 ± 0.40	28.95 ± 0.54	4.00 ± 0.06^ab^	0.41 ± 0.02
	CT(85)	32.19 ± 0.36	1.80 ± 0.05	12.46 ± 0.19	29.35 ± 0.25	4.12 ± 0.03^b^	0.40 ± 0.01
	*P-value*	0.8073	0.4185	0.8504	0.6087	0.0128	0.1185
rs134340637	AA(10)	32.65 ± 0.73	1.78 ± 0.10	12.43 ± 0.40	28.95 ± 0.54	4.00 ± 0.06^ab^	0.41 ± 0.02
	GG(220)	32.24 ± 0.33	1.75 ± 0.05	12.54 ± 0.17	29.44 ± 0.22	4.06 ± 0.03^b^	0.38 ± 0.01
	AG(85)	32.19 ± 0.36	1.80 ± 0.05	12.46 ± 0.19	29.35 ± 0.25	4.12 ± 0.03^a^	0.40 ± 0.01
	*P-value*	0.8073	0.4185	0.8504	0.6087	0.0128	0.1185
rs41919992	CC(157)	32.21 ± 0.35	1.79 ± 0.05	12.67 ± 0.18	29.68 ± 0.24^A^	4.05 ± 0.03	0.39 ± 0.01
	TT(24)	31.62 ± 0.54	1.80 ± 0.08	12.69 ± 0.29	30.64 ± 0.39^C^	4.02 ± 0.04	0.38 ± 0.02
	CT(133)	32.42 ± 0.35	1.73 ± 0.05	12.45 ± 0.18	28.89 ± 0.24^B^	4.05 ± 0.03	0.38 ± 0.01
	*P-value*	0.2037	0.2115	0.2499	<.0001	0.8456	0.7406
rs133498277	CC(157)	32.22 ± 0.35	1.80 ± 0.05	12.67 ± 0.18	29.71 ± 0.24^A^	4.05 ± 0.03	0.39 ± 0.01
	TT(23)	31.57 ± 0.54	1.82 ± 0.08	12.59 ± 0.29	30.62 ± 0.39^C^	4.06 ± 0.04	0.38 ± 0.02
	CT(134)	32.44 ± 0.35	1.74 ± 0.05	12.47 ± 0.18	28.92 ± 0.24^B^	4.05 ± 0.03	0.38 ± 0.01
	*P-value*	0.1847	0.2131	0.3740	<.0001	0.9503	0.8353
rs41919984	CC(157)	31.54 ± 0.53	1.79 ± 0.07	12.64 ± 0.29	30.68 ± 0.38^A^	4.06 ± 0.04	0.38 ± 0.02
	TT(24)	32.16 ± 0.34	1.80 ± 0.05	12.62 ± 0.18	29.75 ± 0.24^C^	4.08 ± 0.03	0.39 ± 0.01
	TC(134)	32.41 ± 0.34	1.73 ± 0.05	12.39 ± 0.17	28.88 ± 0.23^B^	4.08 ± 0.03	0.39 ± 0.01
	*P-value*	0.1507	0.2088	0.2673	<.0001	0.8309	0.6460
rs41919985	AA(25)	31.55 ± 0.53	1.8 ± 0.07	12.52 ± 0.29	30.59 ± 0.38^A^	4.07 ± 0.04	0.38 ± 0.02
	GG(157)	32.16 ± 0.34	1.80 ± 0.05	12.61 ± 0.18	29.75 ± 0.24^A^	4.08 ± 0.03	0.39 ± 0.01
	GA(133)	32.41 ± 0.34	1.73 ± 0.05	12.41 ± 0.17	28.88 ± 0.23^B^	4.08 ± 0.03	0.39 ± 0.01
	*P-value*	0.1446	0.1888	0.3716	<.0001	0.9818	0.6229
rs41919986	CC(155)	32.11 ± 0.34	1.80 ± 0.05	12.67 ± 0.18	29.77 ± 0.24^A^	4.05 ± 0.03	0.39 ± 0.01
	TT(25)	31.53 ± 0.53	1.81 ± 0.07	12.54 ± 0.29	30.60 ± 0.38^A^	4.05 ± 0.04	0.38 ± 0.02
	CT(132)	32.38 ± 0.35	1.74 ± 0.05	12.50 ± 0.18	28.88 ± 0.24^B^	4.03 ± 0.03	0.38 ± 0.01
	*P-value*	0.1502	0.1425	0.4945	<.0001	0.7292	0.6757
Locus	Genotypes	C14INDEX	C16INDEX	C18INDEX	SFA	UFA	SFA/UFA
rs136947640	CC(248)	7.62 ± 0.26	5.15 ± 0.12	69.96 ± 0.52	61.45 ± 0.31	36.89 ± 0.28	1.70 ± 0.04
	TT(2)	6.69 ± 1.19	5.44 ± 0.54	71.10 ± 2.50	60.67 ± 1.52	37.49 ± 1.39	1.62 ± 0.20
	CT(64)	7.62 ± 0.31	5.35 ± 0.14	70.06 ± 0.63	61.09 ± 0.38	37.08 ± 0.34	1.68 ± 0.05
	*P-value*	0.7344	0.1582	0.8863	0.4488	0.7360	0.8360
rs41919999	CC(64)	7.39 ± 0.31	5.27 ± 0.14	70.57 ± 0.63	61.22 ± 0.38	37.11 ± 0.35	1.67 ± 0.05
	TT(88)	7.84 ± 0.30	5.22 ± 0.14	70.18 ± 0.60	60.92 ± 0.36	37.43 ± 0.33	1.66 ± 0.05
	CT(162)	7.73 ± 0.26	5.20 ± 0.12	70.13 ± 0.53	61.29 ± 0.31	36.96 ± 0.29	1.69 ± 0.04
	*P-value*	0.2796	0.8450	0.7036	0.4242	0.2017	0.7246
rs132865003	CC(220)	7.74 ± 0.26	5.16 ± 0.12	70.10 ± 0.51	61.31 ± 0.31	37.02 ± 0.28	1.69 ± 0.04
	TT(10)	7.36 ± 0.57	5.17 ± 0.26	70.04 ± 1.19	61.77 ± 0.72	36.54 ± 0.66	1.71 ± 0.10
	CT(85)	7.60 ± 0.28	5.30 ± 0.13	70.22 ± 0.57	61.17 ± 0.34	37.06 ± 0.31	1.68 ± 0.05
	*P-value*	0.6364	0.2951	0.9567	0.6591	0.7296	0.9378
rs134340637	AA(10)	7.36 ± 0.57	5.17 ± 0.26	70.04 ± 1.19	61.77 ± 0.72	36.54 ± 0.66	1.71 ± 0.10
	GG(220)	7.74 ± 0.26	5.16 ± 0.12	70.10 ± 0.51	61.31 ± 0.31	37.02 ± 0.28	1.69 ± 0.04
	AG(85)	7.60 ± 0.28	5.30 ± 0.13	70.22 ± 0.57	61.17 ± 0.34	37.06 ± 0.31	1.68 ± 0.05
	*P-value*	0.6364	0.2951	0.9567	0.6591	0.7296	0.9378
rs41919992	CC(157)	7.66 ± 0.27	5.30 ± 0.12^a^	70.06 ± 0.55	61.03 ± 0.33^A^	37.27 ± 0.30^A^	1.67 ± 0.04
	TT(24)	7.50 ± 0.42	5.40 ± 0.19^ab^	70.71 ± 0.87	60.33 ± 0.52^A^	38.22 ± 0.48^A^	1.60 ± 0.07
	CT(133)	7.56 ± 0.28	5.08 ± 0.13^b^	69.86 ± 0.55	61.82 ± 0.33^B^	36.44 ± 0.30^B^	1.72 ± 0.04
	*P-value*	0.8491	0.0190	0.5297	0.0004	<.0001	0.0612
rs133498277	CC(157)	7.58 ± 0.27	5.31 ± 0.12^a^	70.07 ± 0.54	61.05 ± 0.32^A^	37.31 ± 0.29^A^	1.67 ± 0.04
	TT(23)	7.42 ± 0.42	5.45 ± 0.19^a^	70.84 ± 0.87	60.29 ± 0.53^A^	38.25 ± 0.48^A^	1.60 ± 0.07
	CT(134)	7.48 ± 0.27	5.10 ± 0.12^b^	69.81 ± 0.54	61.84 ± 0.32^B^	36.46 ± 0.30^B^	1.73 ± 0.04
	*P-value*	0.8410	0.0178	0.4115	0.0004	<.0001	0.0617
rs41919984	CC(157)	7.53 ± 0.42	5.37 ± 0.19^ab^	70.82 ± 0.86	60.27 ± 0.52^A^	38.29 ± 0.47^A^	1.60 ± 0.07^a^
	TT(24)	7.77 ± 0.27	5.32 ± 0.12^b^	70.24 ± 0.54	60.91 ± 0.32^A^	37.40 ± 0.29^A^	1.66 ± 0.04^ab^
	TC(134)	7.61 ± 0.27	5.08 ± 0.12^a^	69.95 ± 0.53	61.77 ± 0.32^B^	36.48 ± 0.29^B^	1.72 ± 0.04^b^
	*P-value*	0.6745	0.0154	0.4795	0.0002	<.0001	0.0450
rs41919985	AA(25)	7.51 ± 0.41	5.38 ± 0.19^ab^	70.97 ± 0.85	60.31 ± 0.51^A^	38.23 ± 0.47^A^	1.60 ± 0.07^a^
	GG(157)	7.77 ± 0.27	5.32 ± 0.12^b^	70.24 ± 0.54	60.91 ± 0.32^A^	37.4 ± 0.29^A^	1.66 ± 0.04^ab^
	GA(133)	7.61 ± 0.27	5.07 ± 0.12^a^	69.93 ± 0.53	61.77 ± 0.32^B^	36.47 ± 0.29^B^	1.72 ± 0.04^b^
	*P-value*	0.6642	0.0122	0.3517	0.0002	<.0001	0.0472
rs41919986	CC(155)	7.61 ± 0.27	5.34 ± 0.12^A^	70.09 ± 0.54	60.97 ± 0.32^A^	37.37 ± 0.29^A^	1.66 ± 0.04^ab^
	TT(25)	7.43 ± 0.41	5.45 ± 0.19^A^	70.92 ± 0.85	60.33 ± 0.51^A^	38.22 ± 0.47^A^	1.60 ± 0.07^b^
	CT(132)	7.49 ± 0.27	5.09 ± 0.12^B^	69.71 ± 0.54	61.86 ± 0.32^B^	36.41 ± 0.29^B^	1.73 ± 0.04^a^
	*P-value*	0.7937	0.0074	0.2439	0.0001	<.0001	0.0393

Notes: *P-value* refers to the results of the association analysis between each SNP and milk fatty acid traits. Different letter (small letters: *P* < 0.05; capital letters: *P* < 0.01) superscripts (adjusted value after correction for multiple testing) indicate significant differences among the genotypes

The effects of the three genotyped polymorphisms in *PPARGC1A*, *ABCG2* and *IGF1* on 16 milk fatty acid compositions are shown in Table 5. SNP rs109579682 in *PPARGC1A* was significantly associated with eight milk fatty acid traits, such as C10:0 (*P* = 0.0251), C12:0 (*P* = 0.0340), C14:0 (*P* = 0.0188), C16:1 (*P* = 0.0401), C18:1n9c (*P* = 0.0015), C16 index (*P* = 0.0010), SFA (*P* = 0.0065) and UFA (*P* = 0.0038). Correspondingly, the CC genotype was the dominant type for saturated fatty acids (C10:0, C12:0, C14:0 and SFA), and the TT genotype was dominant for unsaturated fatty acids (C16:1, C18:1n9c, C16 index and UFA).

**Table 5:** Associations of SNPs of *PPARGC1A, ABCG2 and IGF1* genes with milk medium-chain fatty acids (MCFAs) in Chinese Holstein cattle (LSM ± SE)

Gene	Locus	Genotypes	C10:0	C12:0	C14:0	C14:1		
*PPARGC1A*	rs109579682	CC(27)	2.10 ± 0.06^ab^	2.66 ± 0.07^a^	9.50 ± 0.13^a^	0.77 ± 0.03		
		TT(170)	1.94 ± 0.08^b^	2.42 ± 0.11^b^	9.19 ± 0.19^ab^	0.79 ± 0.05		
		CT(147)	2.13 ± 0.06^a^	2.62 ± 0.08^ab^	9.30 ± 0.13^b^	0.78 ± 0.03		
		*P-value*	0.0251	0.034	0.0188	0.8281		
*ABCG2*	rs137757790	AA(115)	2.13 ± 0.06	2.67 ± 0.08	9.58 ± 0.13^A^	0.78 ± 0.04		
		CC(85)	2.06 ± 0.06	2.58 ± 0.08	9.21 ± 0.14^B^	0.76 ± 0.04		
		CA(145)	2.12 ± 0.06	2.64 ± 0.08	9.50 ± 0.13^A^	0.78 ± 0.03		
		*P-value*	0.2206	0.3385	0.0026	0.7772		
*IGF1*	rs109763947	CC(58)	2.06 ± 0.07^a^	2.64 ± 0.09	9.47 ± 0.15	0.77 ± 0.04		
		TT(100)	2.19 ± 0.06^b^	2.72 ± 0.08	9.57 ± 0.14	0.77 ± 0.04		
		CT(187)	2.10 ± 0.06^ab^	2.60 ± 0.07	9.42 ± 0.13	0.78 ± 0.03		
		*P-value*	0.0342	0.0764	0.2805	0.9454		
Gene	Locus	Genotypes	C16:0	C16:1	C18:0	C18:1n9c	C18:2n6c	CLA
*PPARGC1A*	rs109579682	CC(27)	32.44 ± 0.33	1.71 ± 0.05^a^	12.61 ± 0.17	29.19 ± 0.22^A^	4.07 ± 0.03	0.39 ± 0.01
		TT(170)	32.40 ± 0.51	1.82 ± 0.07^ab^	12.59 ± 0.27	30.08 ± 0.36^B^	4.08 ± 0.04	0.37 ± 0.02
		CT(147)	31.99 ± 0.34	1.79 ± 0.05^b^	12.59 ± 0.17	29.74 ± 0.23^B^	4.07 ± 0.03	0.38 ± 0.01
		*P-value*	0.1541	0.0401	0.9845	0.0015	0.9515	0.6788
*ABCG2*	rs137757790	AA(115)	32.45 ± 0.35	1.72 ± 0.05	12.52 ± 0.18	29.12 ± 0.24^A^	4.05 ± 0.03	0.37 ± 0.01
		CC(85)	31.99 ± 0.37	1.71 ± 0.05	12.79 ± 0.19	29.91 ± 0.26^B^	4.08 ± 0.03	0.38 ± 0.01
		CA(145)	32.33 ± 0.33	1.76 ± 0.05	12.48 ± 0.17	29.50 ± 0.22^AB^	4.07 ± 0.03	0.39 ± 0.01
		*P-value*	0.3251	0.3431	0.1475	0.0048	0.6085	0.2071
*IGF1*	rs109763947	CC(58)	32.29 ± 0.39	1.81 ± 0.05	12.44 ± 0.20	29.42 ± 0.27^AB^	4.08 ± 0.03^A^	0.39 ± 0.01
		TT(100)	32.52 ± 0.36	1.7 ± 0.05	12.62 ± 0.18	29.02 ± 0.25^B^	3.99 ± 0.03^B^	0.38 ± 0.01
		CT(187)	32.19 ± 0.33	1.73 ± 0.05	12.57 ± 0.16	29.70 ± 0.22^A^	4.10 ± 0.03^A^	0.38 ± 0.01
		*P-value*	0.4406	0.0797	0.6613	0.0024	<.0001	0.5835
Gene	Locus	Genotypes	C14INDEX	C16INDEX	C18INDEX	SFA	UFA	SFA/UFA
*PPARGC1A*	rs109579682	CC(27)	7.50 ± 0.26	5.03 ± 0.12^A^	69.81 ± 0.51	61.62 ± 0.30^A^	36.75 ± 0.28^A^	1.70 ± 0.04
		TT(170)	7.89 ± 0.40	5.33 ± 0.18^B^	70.52 ± 0.82	60.75 ± 0.49^B^	37.74 ± 0.45^B^	1.64 ± 0.07
		CT(147)	7.75 ± 0.27	5.33 ± 0.12^B^	70.24 ± 0.53	60.92 ± 0.32^B^	37.38 ± 0.29^B^	1.66 ± 0.04
		*P-value*	0.3099	0.0010	0.4283	0.0065	0.0038	0.2931
*ABCG2*	rs137757790	AA(115)	7.51 ± 0.28	5.05 ± 0.13	69.96 ± 0.55	61.64 ± 0.33^A^	36.67 ± 0.30^a^	1.71 ± 0.04
		CC(85)	7.62 ± 0.29	5.10 ± 0.13	70.00 ± 0.59	60.81 ± 0.35^B^	37.45 ± 0.32^b^	1.65 ± 0.05
		CA(145)	7.57 ± 0.26	5.19 ± 0.12	70.29 ± 0.52	61.33 ± 0.31^AB^	37.11 ± 0.28^ab^	1.68 ± 0.04
		*P-value*	0.8925	0.3350	0.7065	0.0343	0.0266	0.4267
*IGF1*	rs109763947	CC(58)	7.50 ± 0.31	5.32 ± 0.14^a^	70.26 ± 0.62	61.14 ± 0.37^A^	37.10 ± 0.34^AB^	1.67 ± 0.05
		TT(100)	7.5 ± 0.28	4.98 ± 0.13^b^	69.68 ± 0.56	61.88 ± 0.33^B^	36.47 ± 0.30^B^	1.73 ± 0.04
		CT(187)	7.62 ± 0.26	5.13 ± 0.12^ab^	70.31 ± 0.51	61.10 ± 0.30^A^	37.31 ± 0.28^A^	1.66 ± 0.04
		*P-value*	0.8036	0.0239	0.3301	0.009	0.0023	0.1970

Notes: *P-value* refers to the results of the association analysis between each SNP and milk fatty acid traits. Different letter (small letters: *P* < 0.05; capital letters: *P* < 0.01) superscripts (adjusted value after correction for multiple testing) indicate significant differences among the genotypes

For *ABCG2*, SNP rs137757790 was significantly associated with C14:0 (*P* = 0.0026), C18:1n9c (*P* = 0.0048), SFA (*P* = 0.0343) and UFA (*P* = 0.0266). The AA genotype was dominant for saturated fatty acids (C14:0 and SFA), and the CC genotype was dominant for unsaturated fatty acids (C18:1n9c and UFA).

For *IGF1*, SNP rs109763947 was significantly associated with C10:0 (*P* = 0.0342), C18:1n9c (*P* = 0.0024), C18:2n6c (*P* < 0.0001), C16 index (*P* = 0.0239), SFA (*P* = 0.0090) and UFA (*P* = 0.0023). The homozygous genotype of TT was the dominant type for saturated fatty acids (C10:0 and SFA), and the heterozygous genotype of CT was the dominant type for unsaturated fatty acids (C18:1n9c, C16 index, C18:2n6c and UFA).

Additionally, the significant dominant, additive and allele substitution effects of the significant SNPs on the target milk fatty acid traits were observed (Tables 6 and 7).

**Table 6:** Additive, dominant and allele substitution effects of the nine SNPs on milk fatty acids traits of *FASN* in Chinese Holstein cattle

Locus	Genetic effect	C10:0	C12:0	C14:0	C14:1	C16:0	C16:1	C18:0	C18:1n9c	C18:2n6c	CLA	C14 INDEX	C16 INDEX	C18 INDEX	SFA	UFA	SFA/ UFA
rs136947640	a	−0.052	−0.011	0.315	0.070	−0.039	−0.056	0.169	−0.409	0.147*	0.000	0.462	−0.143	−0.570	0.390	−0.301	0.036
	d	−0.093	−0.082	0.186	0.063	−0.164	0.007	0.127	−0.409	0.244**	0.015	0.470	0.057	−0.465	0.029	−0.113	0.016
	α	−0.125	−0.075	0.461	0.120	−0.167	−0.050	0.269	−0.729	0.339**	0.012	0.831	−0.099	−0.934	0.412	−0.390	0.049
rs41919999	a	0.072*	0.074	0.192**	−0.011	−0.008	0.013	−0.186	−0.193	0.007	0.008	−0.226	0.024	0.194	0.150	−0.160	0.008
	d	0.090*	0.123*	0.080	0.022	−0.111	−0.021	0.012	−0.267	−0.021	−0.003	0.116	−0.041	−0.245	0.224	−0.308	0.022
	α	0.079**	0.084*	0.198**	−0.009	−0.017	0.012	−0.185	−0.214	0.005	0.007	−0.217	0.020	0.175	0.167	−0.183	0.010
rs132865003	a	−0.029	−0.020	0.034	0.024	−0.206	−0.014	0.058	0.246	0.027	−0.012	0.195	−0.009	0.030	−0.230	0.238	−0.011
	d	−0.026	−0.018	0.004	0.006	−0.256	0.035	−0.022	0.162	0.089*	0.005	0.046	0.139	0.157	−0.373	0.274	−0.019
	α	−0.047	−0.032	0.037	0.028	−0.376	0.009	0.043	0.354	0.087	−0.009	0.225	0.083	0.135	−0.479	0.421	−0.024
rs134340637	a	0.029	0.020	−0.034	−0.024	0.206	0.014	−0.058	−0.246	−0.027	0.012	−0.195	0.009	−0.030	0.230	−0.238	0.011
	d	−0.026	−0.018	0.004	0.006	−0.256	0.035	−0.022	0.162	0.089*	0.005	0.046	0.139	0.157	−0.373	0.274	−0.019
	α	0.047	0.032	−0.037	−0.028	0.376	−0.009	−0.043	−0.354	−0.087	0.009	−0.225	−0.083	−0.135	0.479	−0.421	0.024
rs41919992	a	−0.001	0.042	−0.022	0.008	0.297	−0.006	−0.007	−0.482**	0.012	0.004	0.080	−0.049	−0.326	0.350	−0.475*	0.033
	d	0.139**	0.250**	0.418**	0.030	0.510	−0.064	−0.235	−1.266**	0.010	−0.002	−0.022	−0.271**	−0.524	1.142**	−1.309**	0.091
	α	0.058	0.148*	0.155	0.021	0.513	−0.033	−0.107	−1.018**	0.016	0.003	0.071	−0.163	−0.547	0.834*	−1.029**	0.071
rs133498277	a	−0.011	0.032	−0.048	0.006	0.324	1.796	0.041	−0.453*	−0.003	0.003	0.081	−0.068	−0.384	0.376	−0.471*	0.033
	d	0.120**	0.229**	0.373**	0.027	0.537	1.740	−0.164	−1.249**	−0.008	−0.002	−0.026	−0.284**	−0.639	1.167**	−1.318**	0.092
	α	−0.062	−0.066	−0.208**	−0.005	0.094	1.819	0.111	0.080	0.000	0.004	0.092	0.053	−0.111	−0.122	0.092	−0.006
rs41919984	a	0.009	−0.036	0.048	−0.010	−0.308	−0.003	0.011	0.463*	−0.012	−0.005	−0.121	0.027	0.292	−0.323	0.445*	−0.030
	d	0.138**	0.258**	0.409**	0.027	0.559	−0.059	−0.233	−1.333**	0.012	−0.003	−0.042	−0.268**	−0.577	1.183**	−1.372**	0.095*
	α	0.067*	0.073	0.220**	0.001	−0.072	−0.028	−0.087	−0.100	−0.006	−0.006	−0.138	−0.086	0.049	0.176	−0.135	0.010
rs41919985	a	0.020	−0.020	0.087	−0.009	−0.305	0.000	−0.045	0.421*	−0.004	−0.006	−0.130	0.033	0.366	−0.302	0.415	−0.029
	d	0.125**	0.239**	0.361**	0.025	0.560	−0.063	−0.162	−1.290**	0.003	−0.001	−0.030	−0.278**	−0.677	1.164**	−1.344**	0.094*
	α	−0.032	−0.120	−0.064	−0.019	−0.539	0.026	0.022	0.962**	−0.005	−0.005	−0.117	0.150	0.650	−0.790*	0.978**	−0.068
rs41919986	a	−0.025	0.020	−0.073	0.006	0.293	−0.006	0.068	−0.415*	−0.001	0.004	0.092	−0.050	−0.414	0.318	−0.425	0.029
	d	0.122**	0.224**	0.350**	0.024	0.563	−0.072	−0.106	−1.301**	−0.017	−0.004	−0.032	−0.301**	−0.798	1.210**	−1.390**	0.097*
	α	0.026	0.113	0.072	0.016	0.528	−0.036	0.024	−0.957**	−0.008	0.002	0.079	−0.176	−0.746	0.822*	−1.004**	0.070

Note: a means additive effect; d means dominant effect; α means allele substitution effect. The asterisk (*) means the additive, dominant or allele substitution effect of the locus indicated differ at *P* < 0.05 and the asterisk (**) means the additive, dominant or allele substitution effect of the locus indicated differ at *P* < 0.01

**Table 7:** Additive, dominant and allele substitution effects of the SNPs on milk fatty acids traits of *PPARGC1A, ABCG2 and IGF1* in Chinese Holstein cattle

Gene	Locus	Genetic effect	C10:0	C12:0	C14:0	C14:1	C16:0	C16:1	C18:0	C18:1n9c	C18:2n6c	CLA	C14 INDEX	C16 INDEX	C18 INDEX	SFA	UFA	SFA/UFA
*PPARGC1A*	rs109579682	a	0.081*	0.120**	0.159*	−0.012	0.019	−0.052	0.008	−0.445**	−0.005**	0.006	−0.193	−0.153*	−0.356	0.436*	−0.496*	0.034
		d	0.107*	0.080	−0.049	−0.000	−0.435	0.025	−0.016	0.108	−0.001**	0.003	0.052	0.150	0.075	−0.270	0.134	−0.009
		α	0.036	0.086*	0.179**	−0.012	0.200	−0.062*	0.015	−0.490**	−0.005*	0.005	−0.215	−0.216**	−0.387	0.549**	−0.552**	0.037
*ABCG2*	rs137757790	a	0.039	0.047	0.185**	0.011	0.232	0.004	−0.136	−0.392**	−0.013	−0.003	−0.059	−0.025	−0.021	0.413**	−0.390**	0.028
		d	0.031	0.022	0.106	0.008	0.106	0.047	−0.173	−0.020	0.002	0.012	0.005	0.112	0.312	0.104	0.055	0.000
		α	0.041	0.049	0.195**	0.012	0.241	0.008	−0.151	−0.393**	−0.013	−0.002	−0.058	−0.015	0.007	0.422**	−0.385**	0.028
*IGF-1*	rs109763947	a	−0.063*	−0.040	−0.050	−0.004	−0.115	0.055	−0.088	0.201	0.045	0.005	0.003	0.168**	0.290	−0.369*	0.317*	−0.028
		d	−0.026	−0.083	−0.097	0.007	−0.220	−0.023	0.037	0.482**	0.064	−0.006	0.117	−0.023	0.334	−0.414	0.523*	−0.033
		α	−0.060*	−0.030	−0.038	−0.005	−0.088	0.057	−0.093	0.142	0.037	0.006	−0.012	0.171**	0.250	−0.319	0.253	−0.024

Note: a means additive effect; d means dominant effect; α means allele substitution effect. The asterisk (*) means the additive, dominant or allele substitution effect of the locus indicated differ at *P* < 0.05 and the asterisk (**) means the additive, dominant or allele substitution effect of the locus indicated differ at *P* < 0.01

### LD between the SNPs identified in the four candidate genes and our previous GWAS

**Fig. 1:**
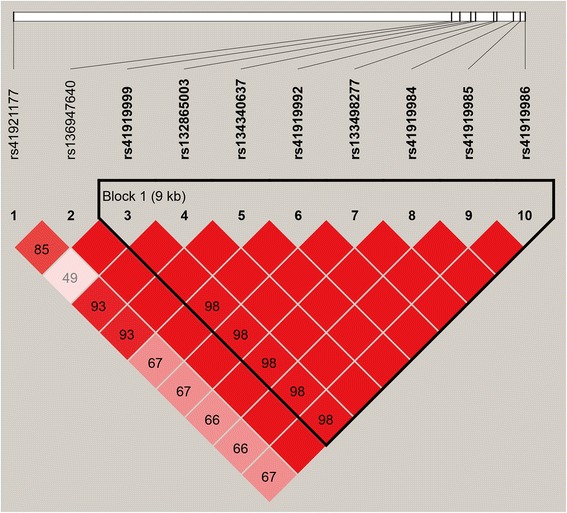
Linkage disequilibrium (LD) plot for 10 SNPs close to or within *FASN*. The values in boxes are pair-wise SNP correlations (D’), bright red boxes without numbers indicate complete LD (D’ = 1). The blocks indicate haplotype blocks and the texts above the horizontal numbers are the SNP names

Pair-wise D’ measures showed that all nine SNPs in *FASN* were highly linked (D’ > 0.9), and one haplotype block comprising eight SNPs was inferred (Fig. 1) in which three haplotypes were formed. The common haplotypes TCGCCTGC, CCGTTCAT and CTACCTGC occurred at a frequency of 54.2 %, 27.8 % and 17.2 %, respectively (Table 8). Most importantly, the significant SNP (rs41921177) identified in our previous GWAS [26] showed strong linkage with the three *FASN* SNPs (rs136947640, rs132865003 and rs134340637). Subsequently, haplotype-based analysis showed significant associations of the haplotypes encompassing the eight *FASN* SNPs (rs41919999, rs132865003, rs134340637, rs41919992, rs133498277, rs41919984, rs41919985 and rs41919986) with C10:0, C12:0, C14:0, C18:1n9c, SFA and UFA (*P* = 0.0204 to *P* < 0.0001; Table 9).

**Table 8:** Main haplotypes and their frequencies observed in the *FASN* gene

*FASN* Haplotypes	SNP3 C > T	SNP4 T > C	SNP5 G > A	SNP6 C > T	SNP7 T > C	SNP8 T > C	SNP9 A > G	SNP10 T > C	Frequency (%)
TCGCCTGC	T	C	G	C	C	T	G	C	54.2
CCGTTCAT	C	C	G	T	T	C	A	T	27.8
CTACCTGC	C	T	A	C	C	T	G	C	17.2

Note: The Ref number of each SNP can be found in the haplotype plot. Also, SNP3 = rs41919999, SNP4 = rs132865003, SNP5 = rs134340637, SNP6 = rs41919992, SNP7 = rs133498277, SNP8 = rs41919984, SNP9 = rs41919985, SNP10 = rs41919986

**Table 9:** Haplotype associations of the eight SNPs in *FASN* with milk production traits in Chinese Holstein cattle (LSM ± SE)

*FASN* haplotypes	C10:0	C12:0	C14:0	C14:1	C16:0	C16:1
H1H1(88)	2.02 ± 0.05^a^	2.54 ± 0.07^A^	9.27 ± 0.12^A^	0.78 ± 0.03	32.45 ± 0.33	1.79 ± 0.05
H2H1(103)	2.23 ± 0.05^b^	2.81 ± 0.06^B^	9.75 ± 0.11^B^	0.80 ± 0.03	32.41 ± 0.29	1.72 ± 0.04
H2H2(24)	2.12 ± 0.09^b^	2.54 ± 0.12^B^	9.53 ± 0.20^AB^	0.76 ± 0.06	31.54 ± 0.53	1.79 ± 0.07
H2H3(28)	2.16 ± 0.08^b^	2.80 ± 0.10^B^	9.95 ± 0.17^B^	0.76 ± 0.05	32.90 ± 0.48	1.76 ± 0.07
H3H1(57)	2.09 ± 0.06^b^	2.60 ± 0.08^B^	9.22 ± 0.14^A^	0.77 ± 0.04	31.84 ± 0.37	1.82 ± 0.05
H3H3(10)	2.16 ± 0.13^b^	2.72 ± 0.17^B^	9.43 ± 0.29^AB^	0.74 ± 0.08	32.59 ± 0.77	1.80 ± 0.11
*P-value*	0.0204	0.0057	0.0001	0.9268	0.2257	0.4522
*FASN* haplotypes	C18:0	C18:1n9c	C18:2n6c	CLA	C14index	C16index
H1H1(88)	12.69 ± 0.18	29.59 ± 0.24^AC^	4.07 ± 0.03	0.38 ± 0.01	7.79 ± 0.26	5.26 ± 0.12
H2H1(103)	12.51 ± 0.16	28.89 ± 0.21^BC^	4.04 ± 0.02	0.37 ± 0.01	7.61 ± 0.23	5.03 ± 0.10
H2H2(24)	12.62 ± 0.29	30.56 ± 0.38^A^	4.05 ± 0.04	0.37 ± 0.02	7.46 ± 0.42	5.36 ± 0.19
H2H3(28)	12.22 ± 0.26	28.31 ± 0.34^B^	4.14 ± 0.04	0.40 ± 0.02	7.16 ± 0.37	5.08 ± 0.17
H3H1(57)	12.65 ± 0.20	29.85 ± 0.27^A^	4.11 ± 0.03	0.39 ± 0.01	7.68 ± 0.29	5.45 ± 0.13
H3H3(10)	12.38 ± 0.41	28.91 ± 0.55^ABC^	4.03 ± 0.06	0.40 ± 0.03	7.23 ± 0.60	5.25 ± 0.27
*P-value*	0.6616	<.0001	0.0792	0.4543	0.7264	0.0548
*FASN* Haplotypes	C18index	SFA	UFA	SFA/UFA		
H1H1(88)	69.95 ± 0.53	61.15 ± 0.32^AB^	37.20 ± 0.29^AB^	1.68 ± 0.04		
H2H1(103)	69.71 ± 0.47	61.87 ± 0.29^A^	36.40 ± 0.26^A^	1.73 ± 0.04		
H2H2(24)	70.75 ± 0.86	60.39 ± 0.52^AB^	38.15 ± 0.47^B^	1.61 ± 0.07		
H2H3(28)	69.85 ± 0.77	62.20 ± 0.46^AB^	35.99 ± 0.42^A^	1.75 ± 0.06		
H3H1(57)	70.25 ± 0.60	60.69 ± 0.36^B^	37.55 ± 0.33^B^	1.64 ± 0.05		
H3H3(10)	70.11 ± 1.24	61.75 ± 0.74^AB^	36.54 ± 0.68^AB^	1.71 ± 0.10		
*P-value*	0.8619	0.0025	<.0001	0.2846		

Notes: *P-value* refers to the results of the association analysis between each haplotype and milk fatty acid traits. Different letter (small letters: *P* < 0.05; capital letters: *P* < 0.01) superscripts (adjusted value after correction for multiple testing) indicate significant differences among the haplotypes. H1 = TCGCCTGC, H2 = CCGTTCAT, H3 = CTACCTGC

**Fig. 2:**
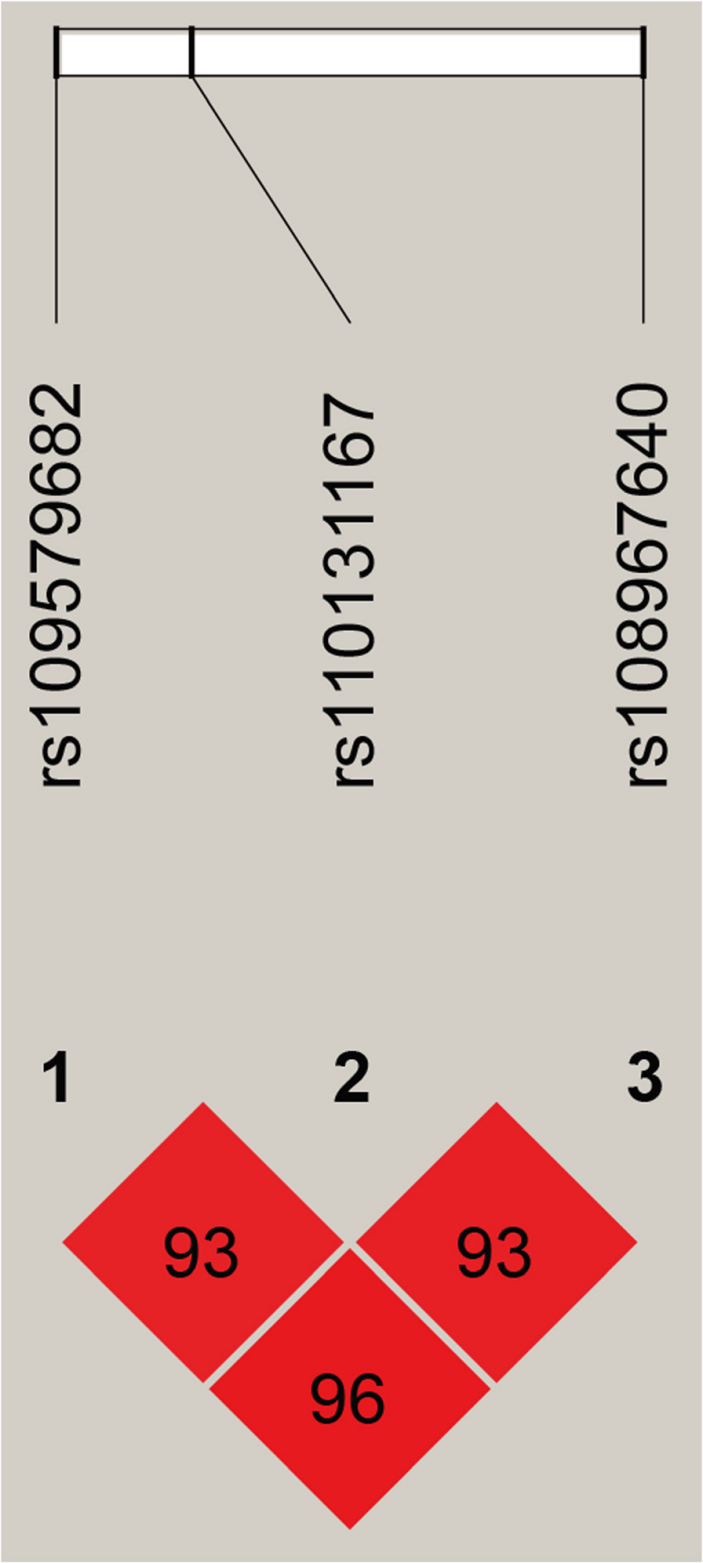
Linkage disequilibrium (LD) plot for three SNPs in *PPARGC1A*. The values in boxes are pair-wise SNP correlations (D’), the brighter shade of red indicates higher linkage disequilibrium

Strong linkage among the two significant SNPs (rs110131167 and rs108967640) detected in our previous GWAS [26] and the SNP (rs109579682) in *PPARGC1A* was also observed (D’ > 0.9, Fig. 2). However, no LD was observed between the SNPs located in the *ABCG2* and *IGF1* genes.

## Discussion

Information on the effects of DNA polymorphisms on milk fatty acid composition is scarce, because milk fatty acid composition data, unlike those of milk fat percentage and fat yield, are not collected routinely in milk recording schemes. Therefore, we attempted to explore the genetic variants of candidate genes identified by our previous GWAS on milk fatty acid composition [26]. In this study, we first investigated the associations between the tested SNPs of *FASN*, *PPARGC1A*, *ABCG2* and *IGF1* and milk fatty acid traits in Chinese Holstein cows.

In our previous GWAS, the SNP rs41921177, at a distance of 58,172 bp away from *FASN*, showed significant association with C10:0 (*P* = 8.54E-06), C12:0 (*P* = 1.16E-07) and C14:0 (*P* = 6.01E-06) [26]. As expected, we found that this SNP was also strongly linked with the three SNPs in *FASN* (rs136947640, rs132865003 and rs134340637) that were significantly associated with C18:2n6c. Furthermore, if the haplotype block was defined based on the solid spine of the LD method, one haplotype block was constructed by the above three SNPs plus two SNPs, rs41921177 and rs41919999, that were associated with C10:0, C12:0 and C14:0. Similarly, strong linkages between the two significant SNPs (rs110131167 and rs108967640) for the C18 index, UFA and SFA/UFA identified in our previous GWAS and the SNP (rs109579682) in *PPARGC1A* for UFA and SFA identified in this study were observed. Probably as a result of the limited number of SNPs identified for *ABCG2* and *IGF1*, and the farther distance between SNPs in the previous GWAS and their adjacent SNPs identified for *ABCG2* and *IGF1* in this study, no linkages with the significant SNPs identified in GWAS were observed.

Six out of nine SNPs in *FASN* (rs41919999, rs41919992, rs133498277, rs41919984, rs41919985 and rs41919986) were markedly associated with C10:0, C12:0 and C14:0, and five of these six SNPs (rs41919992, rs133498277, rs41919984, rs41919985 and rs41919986) also showed significant associations with SFA, which suggested that the *FASN* gene mainly affects the medium-long chain saturated fatty acid traits. FASN is a complex, multifunctional enzyme that catalyzes *de novo* biosynthesis of long-chain saturated fatty acids [36] and plays an essential role in the determination of fatty acid synthesis and release of newly synthesized SFAs [37, 38]. In addition, several previous linkage studies [8, 39, 40] and GWA studies [13–15] have reported that the *FASN* gene is located in a quite large region associated with the medium-chain saturated milk fatty acids on BTA19, which is in agreement with our results that the SNPs in *FASN* mainly showed significant associations with C10:0, C12:0 and C14:0. Moreover, the five SNPs (rs41919992, rs133498277, rs41919984, rs41919985 and rs41919986) also showed associations with the C18:1n9c, C16 index and UFA, and three SNPs (rs136947640, rs132865003 and rs134340637) showed associations with C18:2n6, revealing that the *FASN* gene affects the long-chain unsaturated fatty acid traits. The haplotype-based association analysis showed their significant associations with C10:0, C12:0, C14:0, C18:1n9c, SFA and UFA, also confirming the genetic effects of the *FASN* gene on the medium-chain saturated and long-chain unsaturated milk fatty acids. Kim & Ntambi [41] reported that *FASN* is a key gene involved in the pathway for MUFAs synthesis and incorporation into triacylglycerols and phospholipids, which is consistent with our results. However, the effect of *FASN* on PUFAs has not been reported elsewhere.

It was reported that the SNPs in different exons of the *FASN* gene were associated with milk-fat percentage [9] and with the medium- and long-chain fatty acid content of milk [8] and beef [42]. Morris et al. [8] identified five SNPs in *FASN*, including the non-synonymous SNP, rs41919985, observed in this study, which had been reported in different studies. The allele frequency of rs41919985 A (0.29) in our population is lower than that reported in Friesian and Jersey cattle (0.31 and 0.13, respectively) [8], 0.53 in Dutch Holstein–Friesian population [43] and 0.62 in Angus beef cattle [42]. Morris et al. [8] also reported that rs41919985 affected the C18:1cis9 and the total index, while other SNPs in *FASN* affected C14:0 and C18:2, which were consistent with our findings. Associations of the rs41919985 G allele with higher C14:0 and lower C18:1cis9 were also found in beef cattle [42]. Abe et al. [44] revealed that the *FASN* gene had a significant effect on the fatty acid composition of backfat, intramuscular and intermuscular fat in an F2 population from Japanese Black and Limousin cattle. For all nine significant SNPs in *FASN*, the heterozygous genotypes were associated with a higher proportion of milk SFAs, while the homozygous genotypes were associated with much higher levels of long-chain MUFAs and PUFAs. Thus, decreasing the number of individuals with heterozygous genotypes for these target SNPs in *FASN* will be beneficial to produce high-quality milk with a high proportion of unsaturated fatty acids (UFAs).

PPARGC1A is involved in mammary gland metabolism, and the expression of *PPARGC1A* correlates with milk fat content [45]. Moreover, it is a key factor in energy metabolism and plays a central role in thermogenesis, gluconeogenesis, glucose transport and β-oxidation of fatty acids [46]. The finding that PPAR agonists are able to increase stearoyl-CoA desaturase (*SCD*) mRNA levels in humans, mice and rats suggested that PPARs are able to regulate *SCD* [47]. As the SCD enzyme is involved in the desaturation of saturated fatty acids into cis9-unsaturated fatty acids, PPARs might have an effect on unsaturation indices via their regulation of *SCD* [43]. Our findings supported the above research that *PPARGC1A* was significantly associated with the C16 index. In our study, *PPARGC1A* mainly affected medium-chain saturated fatty acids and long-chain unsaturated fatty acids. Only a few studies have reported associations between *PPARGC1A* and milk fatty acid composition [13, 43]. Schennink et al. [43] found that one SNP in *PPARGC1A*, c.1790 + 514G > A, was associated with the C16:1 and C16 index, and Bouwman et al. [13] reported another significant SNP associated with C16:1, which are in agreement with the results in this study that rs109579682 in *PPARGC1A* is associated significantly with the C16:1 and C16 index. The significant associations between *PPARGC1A* c.1790 + 514G > A and the C14:1, C14 index, and C18 index [43] were not found in this study. The conflicting findings could be explained by the two different genetic backgrounds of the studied populations or by the different number of individuals included in each study. Phenotypic data were available from 1,905 cows in the study reported by Schennink et al. [43], while 346 cows were available in our study.

The bovine *ABCG2* gene is located in the narrow region of chromosome 6 (BTA6), harboring a QTL with a large impact on milk production traits [48, 49]. The ABCG2 protein is responsible for the secretion of xenobiotics and some quantitatively minor nutrients, such as vitamin K3 or cholesterol, into milk [50, 51]. The insulin-like growth factor (IGF) signaling pathway plays a crucial role in the regulation of growth and development of mammals. Liang et al. [52] reported that *IGF1* stimulates *de novo* fatty acid biosynthesis by Schwann cells during myelination. For *ABCG2* and *IGF1*, most studies focused on investigating the association between the identified SNPs in these two genes and milk fat traits [4, 53–58], while limited studies on their association with milk fatty acid composition have been reported [13]. Bouwman et al. [13] reported that one QTL region underlying the *ABCG2* gene showed significant effects on C12:1, C14:1 and C16:1. No association between *IGF1* and milk fatty acids composition has been reported. Further studies will be necessary to confirm our results in different cattle population and to elucidate the mechanisms underlying the association found in this study.

## Conclusions

In this study, we not only confirmed the deduction that the significant SNPs close to the *FASN* and *PPARGC1A* genes identified in our previous GWAS were strongly linked with the key mutations in these two candidate genes, but also presented a link of several variants of *FASN*, *PPARGC1A*, *ABCG2* and *IGF1* with milk fatty acid traits. In particular, *FASN* and *PPARGC1A* mainly affected medium-chain saturated fatty acids and long-chain unsaturated fatty acids. Our findings regarding genes and polymorphisms responsible for the variation of milk fatty acids composition provide useful information that can be combined with breeding programs to tailor the fatty acid content in cow’s milk.

## Abbreviations

ABCG2, ATP-binding cassette, sub-family G, member 2; CAD, coronary artery disease; FASN, fatty acid synthase; GWAS, genome-wide association study; IACUC, institutional animal care and use committee; IGF1, insulin-like growth factor 1; LD, linkage disequilibrium; LDL, low density lipoprotein; MUFA, monounsaturated fatty acid; PPARGC1A, peroxisome proliferator-activated receptor gamma, coactivator 1 alpha; PUFA, polyunsaturated fatty acid; QTL, quantitative trait locus; SCD, stearoyl-CoA desaturase; SFA, saturated fatty acid; UFA, unsaturated fatty acids; UTR, untranslated region.

## Additional file

Additional file 1: Table S1. Primers used to identify SNPs in the *FASN* gene. (PDF 104 kb)
