# Supplementary material for: Genetic effects of FASN, PPARGC1A, ABCG2 and IGF1 revealing the association with milk fatty acids in a Chinese Holstein cattle population based on a post genome-wide association study
Source: BMC Genet. 2016 Jul 28;17:110. doi: 10.1186/s12863-016-0418-x (PMC4963957; doi:10.1186/s12863-016-0418-x)
Supplement: Additional file 1: Table S1. — Primers used to identify SNPs in the FASN gene. (PDF 104 kb) [file 12863_2016_418_MOESM1_ESM.pdf]

Additional file1: Table S1. Primers used to identify SNPs in the *FASN* gene

Serial no	Primer name	Primer sequences	Product size	Annealing temperature
1	Exon-1	L-TCTTTGTTGCAGGGCTTTCT<br>R-TAGCCCAGAGCCTTCTGGTA	499 bp	59 ℃
2	Exon-2	L-AAGCTGGTTTGACTCCCTCA<br>R-CATATTGTGTGCCTGCTTGG	471 bp	59 ℃
3	Exon-3	L-GGAGAATCCCATGGACAGAA<br>R-CTGGAAGGGTCTCAGCTGTC	454 bp	59 ℃
4	Exon-4	L-GCCCTTTTGTTTCTGACTCG<br>R-TGATGCATCGAAAAACTTGC	423 bp	60 ℃
5	Exon-5	L-TTCGATGCATCAGGTGAGAG<br>R-CAACGTACTCCAGGGTCTCC	427 bp	58 ℃
6	Exon-6/7	L-GATGGCTGCAAAGAGAAAGG<br>R-AATGTCTGGCCCTGTGAAAC	615 bp	59 ℃
7	Exon-8	L-GCTTTGGGGGAGAATGAAAT<br>R-CGGAGCAGATGAACCAGAGT	615 bp	60 ℃
8	Exon-9/10	L-CGCCCACTCTGGTTCATC<br>R-AGCGCCAACACAGAAAGG	696 bp**	58 ℃
9	Exon-11/12	L-CCTTTGAGGCTCTCTTCTGC<br>R-CACCAGGTTGTTCACGTTGT	615 bp**	54 ℃
10	Exon-13	L-CATGGCGTTCCACTCCTACT<br>R-TGTCCCTGTGGTCCTTCTTC	545 bp	57 ℃
11	Exon-14	L-GCCGAGTACAACGTGAACAA<br>R-AACTCCACAGGTGGGAACAG	616 bp	58 ℃
12	Exon-15/16	L-AAGCAGACCTCTGGGTGCT<br>R-CATAGCTTGAGCCACCTTCC	630 bp	60 ℃
13	Exon-17/18	L-GAAGGTGGCTCAAGCTATGG<br>R-GCCAGGCTAGGTCTCTTCCT	595 bp**	56 ℃
14	Exon-19	L-GGTCCCACTTTGTGTTTTGG<br>R-TTATTACCACGTCGGCCACT	422 bp	58 ℃
15	Exon-20	L-GTACACGCTGCAGGACAAGA<br>R-GGTAGACCCCACACGTAGGA	406 bp	60 ℃
16	Exon-21	L-CTGAGGCTCTTCCCTGACAT<br>R-CCGATTCTGCAGAGGTCATT	560 bp**	60 ℃
17	Exon-22	L-AAGCCTATTCCATCCCAGGT<br>R-CTGTGAGTTGAGGGGGTCAG	495 bp	60 ℃
18	Exon-23/24	L-CTGACCCCCTCAACTCACAG<br>R-CCCCATCACGGTAGACATTC	627 bp	56 ℃
19	Exon-25	L-GTGGGCTTGGTGAACTGTCT<br>R-TAGGGGGTGACAGAAAGTGG	434 bp	59 ℃
20	Exon-26/27	L-CTTCCCACTGGAACAAGGTG	502 bp**	59 ℃

		R-CCTGACCTCACTGGGAGGAG		
21	Exon-28/29	L-ACCCAACAGTGCTCAGGAAC<br>R-TGCTGCTCAAAGGATGTGTC	674 bp**	57 ℃
22	Exon-30	L-GCCGTGTTTTCCCACTTGT<br>R-GGTCCCCATAGTGAAGGTCA	405 bp	54 ℃
23	Exon-31/32	L-CGGGCTGTGACCTTCACTAT<br>R-CCTGCTCTTCCTCACGTACC	624 bp	58 ℃
24	Exon-33/34	L-GGCAAAGTGGTCATTCAGGT<br>R-GCCTTTGGAGGGCTTCTTAG	654 bp	59 ℃
25	Exon-35	L-GTGGTGAGGACGGCTTTAGA<br>R-GGTCACCCTGTGGGTTCTTA	411 bp	59 ℃
26	Exon-36/37	L-CTTGCACTGGTGTCCAGAGA<br>R-AGACCAGACTCGGAAGACGA	696 bp**	58 ℃
27	Exon-38	L-GGTACAATACCCACCCAGGA<br>R-CCTGCCACAAACACACAGAC	436 bp	59 ℃
28	Exon-39	F-AGAGCTGACGGACTCCACAC<br>R- CTGCATGAAGAAGCACATGG	697 bp**	59 ℃
29	Exon-40/41	L-ACCTGCAGACAAGGGCTAAA<br>R-CAGCTTGTGGTAGAAGGAACG	653 bp	59 ℃
30	Exon-42	F-CTCGCACACCTTCGTGATG<br>R-CACGTTGCCGTGGTAGGTAG	472 bp**	60 ℃

Note: **SNPs are detected.
